# Coupled model for microbial growth and phase mass transfer in pressurized batch reactors in the context of underground hydrogen storage

**DOI:** 10.3389/fmicb.2023.1150102

**Published:** 2023-04-04

**Authors:** Gion Strobel, Birger Hagemann, Christian Truitt Lüddeke, Leonhard Ganzer

**Affiliations:** Institute of Subsurface Energy Systems, Clausthal University of Technology, Clausthal-Zellerfeld, Germany

**Keywords:** underground hydrogen storage, coupled modeling, microbial growth, methanation, mass transfer, hydrogen conversion

## Abstract

A rising interest in a strong hydrogen economy as a part of the future net-zero economy results in an increasing necessity to store hydrogen as a raw material or an energy carrier. Experience and studies show that storing hydrogen in deep underground sites could enable microbial conversion of hydrogen. To predict and examine the loss of hydrogen, laboratory studies, and analysis are essential. A growth model is required to interpret batch or chemostat experiments. With this model, the parameters of microbial growth, and the conversion of hydrogen can be specified. This study presents experiments with methanogens and a hydrogen/carbon dioxide gas mixture performed in batch reactors. Further, the microbial growth was modeled by a double Monod model with hydrogen and carbon dioxide as the limiting substrates. As the amount of carbon dioxide dissolved in the water phase can not be neglected, both phases were considered in the proposed model. The mass-transfer rate between the gas and water phase was implemented by a linear relation including the concentrations in both phases and the mass-transfer coefficient. With the resulting coupled model, it was possible to match the pressure behavior in the reactor and conclude the microbial growth kinetics. Two types of methanogenic species were tested to validate the model. The mass transfer coefficient proves to impact the growth behavior in porous media. The mathematical model and experimental data are necessary to determine the growth rate and yield coefficient.

## 1. Introduction

With the increasing energy demand and the shift toward net-zero, the importance to find suitable long-term energy storage solutions rises. A promising solution is the storage of hydrogen in caverns or porous reservoirs to buffer the fluctuating energy production from renewable energy. Until now, the experience of storing hydrogen in geological formations is limited. However, experience from town gas storage and first field tests in porous reservoirs have delivered promising results. However, the analysis has also shown a potential microbial conversion of hydrogen to different products, such as methane or hydrogen sulfide (Smigáň et al., 1990; Buzek et al., 1994; Pérez et al., 2016; Bauer, 2017). The conversions lead to losses of hydrogen and could potentially impact the storage operation.

The microbes in porous media use hydrogen, dissolved in the formation water, as a source of energy for their metabolism. Three primary dominant conversion processes are identified during underground hydrogen storage: sulfate reduction, methanation, and acetogenesis. The first two directly impact the gas composition in the storage, whereas the acetogenesis impacts the formation waters compositions and pH value. The sulfate-reduction and the methanation are the most crucial as they result in a high energy loss (methanation) or increase the requirements for health and safety during operation due to the hydrogen-sulfide (*H*_2_*S*) production. Especially the production of *H*_2_*S* is dangerous for the safe storage operation as it is a toxic gas.

The growth and metabolisms of microbes can be measured in laboratories by batch reactor experiments with extensive measurements of cell number versus time and changes in the gas and water composition versus time. For microbial conversion during underground hydrogen storage, batch reactor experiments were performed by Bauer (2017, 2020) and Schwab et al. (2022). Bauer et al. showed a conversion of hydrogen into methane in one sampled porous reservoir (Bauer, 2017). Schwab et al. (2022) examined various caverns in Germany, and the performed reactor experiment indicated a slight conversion of hydrogen into several products. Overall, the performed experiments and their results in reactors have shown to be an adequate indicator of a microbial conversion that needs to be considered for the development of a potential hydrogen storage in the particular reservoir. Faster growth leads to a stronger microbial conversion and a significantly shorter experimental duration. In both experimental investigations, the primary indicator for microbial growth is a measured pressure drop in the reactor. Linking the pressure drop to the growth kinetic parameters and simulating the microbial growth was only partially done by Bauer (2017).

The microbial growth is strongly related to the dissolution of the substrate in the formation water. During hydrogen storage, the substrate is injected via the gas phase. Therefore, it has to dissolve in the water to enable the microbial metabolism. In porous media, this substrate transfer from gas to liquid phase may differ significantly because the contact area between the phases is higher compared to a batch reactor (Kimmel et al., 1991; Grimalt Alemany et al., 2018). For that reason, the mass transfer has been coupled with the growth of microbes.

A general storage operation consists of an injection, a withdrawal, and an idle phase. The injection and production phases can be mimicked in the reactor by loading and unloading the gaseous substrates. However, the challenging part is to mimic the idle phase in the reactor and keep the analysis running at the same time. Probing the liquid and gases during the idle phase leads to changes in the volumetric ratios of gas, water and environmental conditions like temperature and pressure in the reactor. These do not appear in a storage scenario.

Therefore an adequate mathematical model is required to reproduce the growth of microbes based on a limited set of measured data. Standard mathematical models describe the growth inside the liquid phase, such as fermentation processes or bio-degradation, e.g, phosphor degradation or nitrification. However, both phases need to be considered in underground hydrogen storage because the substrate is injected via the gas phase. Furthermore, the growth in the reservoir is limited by the available substrate in the water phase (Hagemann et al., 2016). Therefore the model requires mimicking the main phases of microbial growth: lag phase, exponential growth phase and stationary phase (Chmiel, 2011). The basis of a model approach for a reactor is a growth model for the microbes. Common models like the Monod or Contois model have the capability to mimic all stated growth patterns (Murphy and Ginn, 2000). Mazzeo et al. presented a model for a stirred tank reactor that considered the increased mass-transfer coefficient of gas bubbles. However, the substrate was constantly fed into the reactor, which resulted in a missing idle or storage phase (Mazzeo et al., 2021). Also, Jafari et al. presented a model for batch reactors (Jafari et al., 2020). The model matched the gas compositional changes, but not the absolute pressure in the reactor so that the gas volume was not constant during the experiment.

The goal of this paper is to conduct batch reactor experiments with two different methanogenic archaea strains under the influence of hydrogen and carbon dioxide. Potential microbial growth and hydrogen consumption are observed through pressure measurements. A coupled model including mass transfer between the gaseous and the liquid phase is used to model the expected microbial growth. Relevant microbial growth parameters such as maximum growth rate, yield coefficient, lag time, and half-saturation constants should be obtained from the microbial growth model for the respective methanogenic strains.

## 2. Batch reactor experiments

The batch reactor experiments are used to gather data for the mathematical model. This study focuses only on methanation, where carbon dioxide with hydrogen is converted to water and methane. The so-called Sabatier reaction can be written as follows:


(1) $$\begin{array}{l} CO_{2}+4H_{2}\to CH_{4}+2H_{2}O \end{array}$$

Under the assumption that the majority of moles of hydrogen are present in the gas phase of the reactor, the conversion would lead to a reduction of moles and consequently decrease the pressure in the gas phase. The methanation process is selected instead of the sulfate reduction, as methane is easier to handle in the laboratory than the toxic hydrogen sulfate. Further, identifying active methanogens is simpler than sulfate-reducers as they reflect fluorescence light. This is done to calculate the initial concentration of microbes in some experiments.

### 2.1. Experimental setup and materials

For the experiments, two species are used to collect data for two different growth rates and two temperature settings. Both species are hydrogenotrophic methanogens, which convert hydrogen and carbon dioxide to methane and water (Huber et al., 1982; Goker et al., 2014).

- **Methanothermococcus thermolithotrophicus** (defined as Species 1, DSMZ Nr. 2095); Optimal temperature = 65°C; Optimal NaCl-concentration = 4 %; Optimal pH-value = 7; doubling time of population = 55 min
- **Methanolacinia petrolearia** (defined as Species 2, DSMZ Nr. 11571); Optimal temperature = 37°C; Optimal NaCl-concentration = 1–3%; Optimal pH-value = 7; doubling time of population = 10 h

Both species were purchased from the DSMZ as living pre-cultivated microbes and delivered in a test tube sealed with a rubber plug. In order to secure optimal growing conditions, culture medium DSMZ 141 was used, which included all important trace elements for the growth. A gas mixture was used as a substrate carrier consisting of 20% carbon dioxide and 80% hydrogen, which is in alignment with Equation 1, the optimal ratio of carbon dioxide and hydrogen for the growth. The used species are strong anaerobes, and therefore the gas mixture has a high purity to minimize the percentage of air inside the gas. Besides the main materials, isopropanol and pure nitrogen were used to clean and pressurize the reactor.

**Figure 1:**
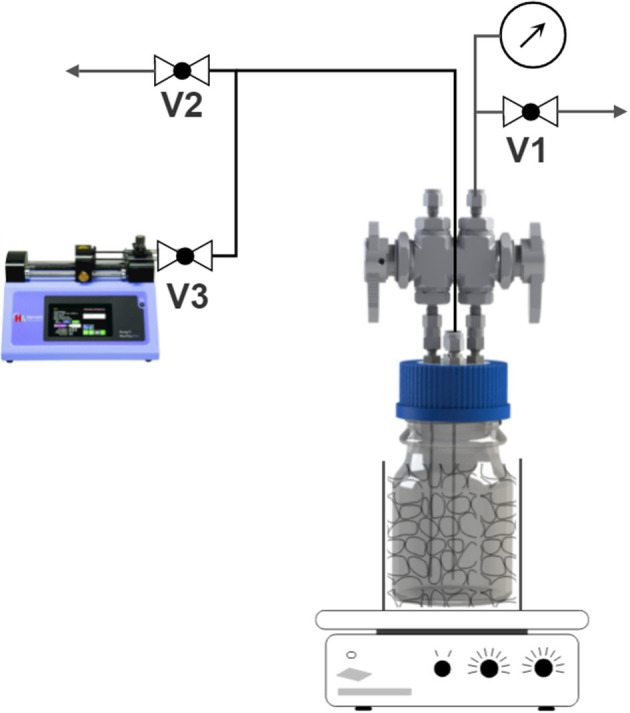
Sketch of the experimental setup, which includes a bottle batch reactor, heating system, pressure sensor, and a syringe pump.

An exemplary reactor is shown in Figure 1. The main part of the reactor is a Duran Pressure Plus bottle with transparent glass, which enables the observation of changes in turbidity due to microbial growth and the flow of gases into the liquid phase. The glass is made of double borosilicate glass 3.3 to withstand a pressure of −1 to 1.5 bar. The total reactor volume is 132.5 ml. A modified GL45 HPLC cap is used to seal the reactor. To prove the tightness of the system, a leakage test is performed before each experiment.

To guarantee the desired optimal temperature for the microbes, the reactor was placed in a metallic bead batch during the experiment, illustrated in Figure 1, which was placed on top of a heating plate. The heating plate controlled the temperature inside the bed, not the reactor. However, preliminary work shows sufficient alignment between the bed and liquid temperature.

The pressure in the gas phase was recorded during each experiment. The pressure sensor used was an absolute pressure sensor (Series PX33 Keller AG) with a measuring range of up to 10 bar. The sensor was connected to the Swagelok valve, which was connected to the gas phase of the reactor. In this way, no water could enter the sensor and block the flow line or the sensor itself.

In one experiment, see Table 1 series 1.1, the liquid inside the reactor was probed three times: at the beginning to get the initial microbial density as an input parameter for the initialization, approximately in the middle, and at the end of the experiment to have a matching parameter for the maximum number of microbes detected. The number of microbes was estimated with the help of a microscope and a microchip to minimize the required volume (Strobel et al., 2021). A syringe pump was used with a syringe to withdraw the liquid probe.

**Table 1:** Overview of the performed experiments relevant for the modeling part; **marked** series are considered for the modeling part.

**Nr**.	**Species**	***P*_0_ [mBar]**	**Temperature [°*C*]**	**Comment and aim of experiment**
1.1	1	600	65	Verify growth of species 1
1.2	1	825	65	Verify growth of species 1 (repetition 1)
1.3	1	1,100	65	Verify growth of species 1 (repetition 2)
2.1	2	550	37	Verify growth of species 2
2.2	2	420	37	Verify growth of species 2 (repetition 1)
2.2	2	960	37	Verify growth of species 2 (repetition 2)
3	1	720	65	Refill reactor five times

Two valves in the modified cap are made of steel, whereas the rest are peek valves. The flowlines are used to connect sensors and syringes to the reactor; they are made of peek as well. Peek has the advantage of being more flexible than steel and provides a low diffusivity.

### 2.2. Experimental procedure and overview

Several experiments were performed, which are summarized in the following list:

As seen in Table 1, the experiments differ in used species, operation pressure and renewing of substrates. The first series of experiments were performed to verify and analyze the functionality and performance of the reactor by conducting two experiments with both species and comparing the pressure drop. The following experiments with different initial pressures were conducted to study the effect of increased solubility on growth, which is out of scope for the modeling and matching part. In the last experimental series, the reactor was refilled several times to study the effect of refilling on the lag phase and the growth rate of microbes.

All experiments were performed in a similar manner:

- First, equipment and tools were cleaned to ensure they are sterile using an autoclave.
- After cleaning, the setup was assembled and flooded with pure nitrogen to check for any leakage and to ensure that the system was completely free from any oxygen.
- In the next step, the culture medium was filled into the bottle reactor under anaerobe conditions. The reactor was filled with 40 ml liquid. During the filling process, the reactor and the pipes were constantly flooded with nitrogen.
- After filling the reactor with culture medium, the reactor was pressurized with nitrogen, and the liquid was checked for its oxygen content (i.e. the solution was checked with Oxygen CHEMets Kit to be below 150 ppb).
- In the following step, a concentrated solution of methanogenic archaea, ca. 7 ml, was injected into the reactor. The microbes were ordered as active living microbes from DSMZ. To transfer the microbes from the tube to the reactor, the procedure provided by the DSMZ was followed accordingly. New (fresh) microbes were used for each experiment so that the culture was always pure. The microbial concentration after the mixing ranged between 1E7 and 1E8 *cells*/*ml*.
- To complete the initialization, the reactor was flooded with the gas mixture of hydrogen and carbon dioxide. The gas was flooded into the liquid phase so that the nitrogen in the gas phase was displaced to the top. After 10–15 min of flushing, the reactor was pressurized to the desired pressure as mentioned in Table 1 with the gas mixture.
- In the last step of the preparation procedure, the reactor was attached to a pressure sensor and placed into the heating system, which heated the reactor to the optimal temperature of the respective microorganism species.

Additional analysis, for instance, probing the liquid, was done at initialization and during the experiments. The duration of each experiment was estimated based on the purpose of the investigation and the pressure inside the reactor. A stable and low pressure value, compared to the initial pressure, indicated a stop of growth and conversion. In most experiments, the pressure dropped below the atmospheric pressure (−300–450 mBar) and stayed constant.

### 2.3. Experimental results

All experiments show a substantial reduction of absolute pressure in the gas phase. As an example of the data collecting, the results of two experiments are shown in the following figures, as they are relevant for the modeling and simulation part.

**Figure 2:**
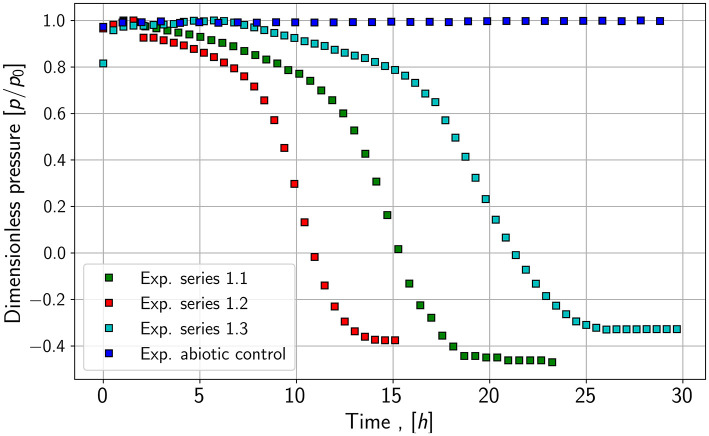
Pressure drop in the gas phase of the reactor for species 1 and a abiotic control.

**Figure 3:**
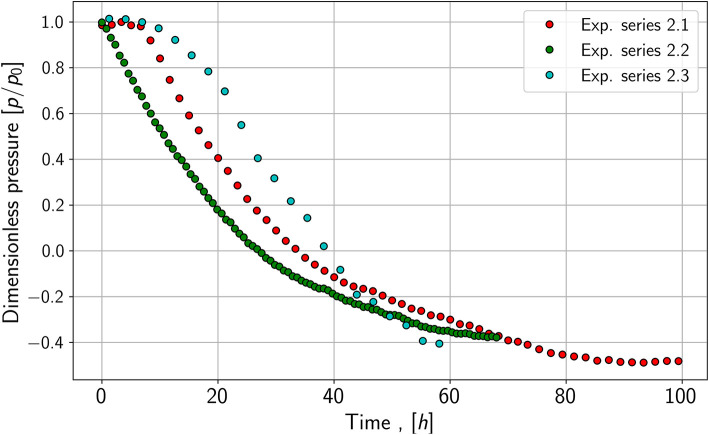
Pressure drop in the gas phase of the reactor for species 2.

As seen in Figure 2, the pressure reduction of species 1, a fast-growing species, is more rapid compared to the pressure reduction of species 2; those growth experiments are shown in Figure 3. It leads to the assumption of differences in growth and conversion rates. Besides the pressure reduction, the microbes' lag phase is also noticeable as the pressure stays constant or only decreases slightly in the first hours of the growth experiments of species 1. Further, the pressure for species 2 does not reach the same end value as species 1. One explanation may be that not all substrates are consumed by the microbes or an inert gas (nitrogen) is still in the system. The control experiments show similar pressure drops for both species. Especially in the case of species 1, the slope of the pressure drop seems to be nearly identical.

Further, the pressure drop inside the reactors acts as an indicator of biotic control. The pressure in the reactor drops below the atmospheric pressure, and the negative values can be seen in the experiment's later stage. In a leaking reactor, the pressure would tend to zero and opening the reactor after the experiments leads to a rapid pressure jump to 0.

**Figure 4:**
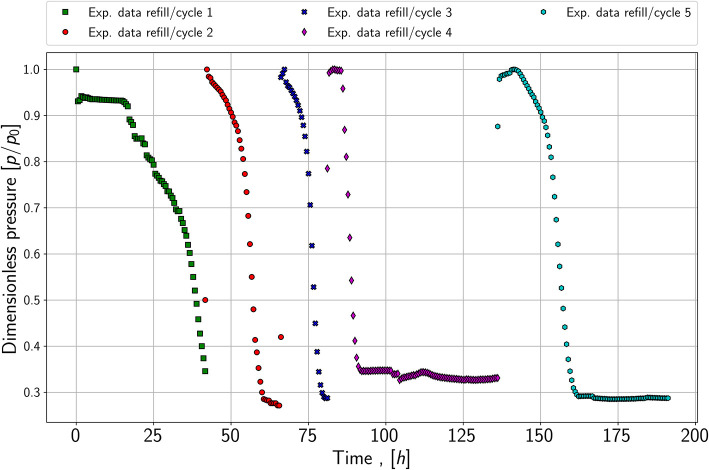
Pressure versus time; refill experiment with five cycles; performed with species 1 and at a pressure of around 1,000 mBar.

The third experiment shows five cycles of growth, plotted in Figure 4. After each growth period, the reactor was refilled with a substrate gas mixture when the pressure reached a constant value. The first important finding is that the lag phase seems to be reduced or it appears to have completely disappeared in the refill cycle; see dark blue and red data points. Furthermore, the slope of the pressure drop appears to be similar in each refill cycle, which may lead to the conclusion that the maximum growth rate is equal in each cycle. This is a second indicator that it is possible to reproduce the growth behavior. Therefore, it is sufficient to match the pressure drop for the growth experiments of each species (series 1.1 and 2.1) and the refill experiment (series 3) in the modeling part.

At the end of the experiments, an increase in the methane concentration and a reduction of hydrogen concentration in the gas were detected by gaschromatography. Due to the low pressure (below atmospheric), the exact concentrations could not be measured. However, the increase in methane is an indicator of a working methanation process in the reactor.

## 3. Coupled model of microbial growth and mass transfer

In order to determine the growth describing kinetic parameters, a 0-D model was developed considering the moles of components in both phases, the transfer of substrates and products between gas and water, and the microbial growth in the liquid phase.

### 3.1. Model approach

A commonly used model to describe the growth of microbes under substrate-limited conditions is the Monod model, which can be extended to account for more than one limited substrate. In the case of methanation, the limited substrates are hydrogen or carbon dioxide, see Equation 1 in the liquid phase of the reactor. Therefore the double Monod model can be written as follows, which describes the growth rate depending on two substrates:


(2) $$\begin{array}{l} \mu_{growth}=\mu_{growth}^{max}\left(\frac{C_{H_{2},w}}{K_{H_{2},w}+C_{H_{2},w}}\right)\left(\frac{C_{CO_{2},w}}{K_{CO_{2},w}+C_{CO_{2},w}}\right) \end{array}$$

where *C*_*H*_2_, *w*_, *C*_*C*_*O*__2_, *w*_ are the mole concentrations of substrates in $\frac{mol}{m^{3}}$ inside the water phase, *K*_*H*_2_, *w*_, *K*_*C*_*O*__2_, *w*_ are the half-saturation constants of the two substrates in $\frac{mol}{m^{3}}$, $\mu_{growth}^{max}$ is the maximal growth rate calculated based on the experiments during the exponential growth phase in $\frac{1}{s}$. The half saturation constant describes the concentration of the respective substrate where the growth rate reaches the half of the maximal growth rate. The half-saturation constant and the maximum growth rate depend on the species and environmental conditions.

In the absence of one or more substrates, the microbes need to maintain their metabolism. Therefore a decay/maintenance coefficient is required to describe this process:


(3) $$\begin{array}{l} \mu_{decay}=-b \end{array}$$

where *b* is a constant decay and maintain coefficient in $\frac{1}{s}$.

As mentioned before and as is also seen in the figures of the experimental results, both microbial species have a lag phase. The lag phase is expressed by a piece-wise function (Wood et al., 1995):


(4) $$\begin{array}{l} \text{λ}=\{\begin{array}{ll} 0 & \;i\text{f }t<t_{L}\\ \frac{t-t_{L}}{t_{E}-t_{L}} & \text{ if }t_{L}⩽\;t\;⩽t_{E}\\ 1 & \text{ if }t>t_{E} \end{array} \end{array}$$

, where *t* is the time of the experiment in *s*, starting with the first contact of the substrate with the liquid, and *t*_*L*_ (*s*) is the end of lag phase until no growth can be noticed in the experiment, and *t*_*E*_ (*s*) is the time when the exponential growth is reached in the experiments.

The change of microbes in the liquid phase can be expressed by combining the Monod model for microbial growth, the decay/maintenance model, and the piece-wise function for the lag phase:


(5) $$\begin{array}{l} \frac{dX_{m}}{dt}=\mu_{growth}^{max}X_{m}{\text{λ}}_{lag}\left(\frac{C_{H_{2},w}}{K_{H_{2},w}+C_{H_{2},w}}\right)\left(\frac{C_{CO_{2},w}}{K_{CO_{2},w}+C_{CO_{2},w}}\right)\;\\ \;-bX_{m}{\text{λ}}_{lag} \end{array}$$

where *X*_*m*_ is the total microbial number in the water phase [*cells*]. The number of microbes stays constant as long as the lag phase function is zero; the microbial number increases until one substrate is depleted and the decay/maintenance term dominates.

Besides the model for the microbial number, the reduction and increase of the main components, namely carbon dioxide, hydrogen, and methane, is modeled. In contrast to the microbial model, the compositional change must be considered for the gas and water phase. The concentration of components inside the water phase (solubility) is usually modeled via the Henry Law, stated in the following equation:


(6) $$\begin{array}{l} H_{n}=C_{w_{n}}/p_{x_{g_{n}}} \end{array}$$

where *C*_*w*_*n*__ is the mol concentration of a component in the liquid phase, *p*_*x*_*g*__ is the partial pressure of that component in the gas phase under equilibrium conditions in *Pa* and *H*_*n*_ is the Henry solubility constant at threshold temperature in $\frac{mol}{m^{3}*Pa}$. To account for the higher temperature inside the reactor, the Henry constant can be corrected with the following approach:


(7) $$\begin{array}{l} H(T)=H{}^{\circ}exp\left[\frac{-{\text{Δ}}_{sol}\cdot H}{R}\cdot \left(\frac{1}{T}-\frac{1}{T{}^{\circ}}\right)\right] \end{array}$$

where −Δ_sol_*H is the enthalpy of dissolution in $\frac{J}{mol}$, *R* is the gas constant in $\frac{J}{mol*K}$, *T* the temperature in *K*, *T*° the threshold temperature. The factor −Δ_*sol*_*H* can be found in the literature for most components (Sander, 2015).

The partial pressure of each component, which is required for the Henry Law, can be written as follows:


(8) $$\begin{array}{l} P_{x}=\frac{N_{g_{n}}}{N_{g_{T}}}*P_{T} \end{array}$$

where *P*_*T*_ is the total pressure of the gas mixture in *Pa*, *N*_*g*_*n*__ is the number of moles for one component in the gas phase, *N*_*g*_*T*__ is the sum of moles in the gas phase, and *P*_*x*_ is the partial pressure of the component required for the Equation 6.

An essential assumption in Henrys law is the equilibrium between the respective concentrations in the gas and water phase. In the reactor, this can be assumed for the initial state but not during the growth of microbes due to the consumption in the water phase. Therefore a mass-transfer model is used to mimic the flow of molecules of the respective component due to the concentration gradient at the interface between water and gas. The model is based on the two-film theory and the “film” between the phase is the resistance against the mass transfer.

As no reaction takes place in the gas phase, the change of the substrates hydrogen and carbon dioxide over time is dominated by the mass transfer and can be written as follows


(9) $$\begin{array}{l} \frac{dN_{{H_{2}}_{g},{CO_{2}}_{g},{CH_{4}}_{g}}}{dt}=-k_{g,w}\left(x_{{H_{2}}_{l},{CO_{2}}_{l},{CH_{4}}_{l}}-x_{{H_{2}}_{li},{CO_{2}}_{li},{CH_{4}}_{li}}\right) \end{array}$$

where *k*_*g, w*_ is the mass transfer coefficient in $\frac{mol}{s}$ for gas and water, which is equal for all components (hydrogen, carbon dioxide, and methane) and *N*_*g*_ is the number of moles in the gas phase. The subscript _*li*_ indicates the concentration in the liquid phase when it would be in equilibrium with the gas phase.

In order to link the production of microbial biomass with the reduction of substrates and increase in products during the methanation metabolism, the yield coefficient is introduced. The yield coefficient describes the amount of biomass produced for one mole of the respective component with the unit $\frac{1}{mol}$. Finally, combining the mass transfer and the microbial reaction, the following set of equations can be written for hydrogen, carbon dioxide and methane inside the liquid phase.


(10) $$\begin{array}{l} \frac{dN_{H_{2}{}_{l},CO_{2}{}_{l},CH_{4}{}_{l}}}{dt}=\frac{1}{Y_{H_{2}}}\zeta_{H_{2},CO_{2},CH_{4}}(\mu_{growth}^{max}X_{m}{\text{λ}}_{lag}\;\\ \;\left(\frac{C_{H_{2},w}}{K_{H_{2},w}+C_{H_{2},w}}\right)\left(\frac{C_{CO_{2},w}}{K_{CO_{2},w}+C_{CO_{2},w}}\right)\\ \;+k_{g,w}(x_{H_{2}{}_{l},CO_{2}{}_{l},CH_{4}{}_{l}}-x_{H_{2}{}_{li},CO_{2}{}_{li},CH_{4}{}_{li}}) \end{array}$$

where ζ describes the stoichiometric coefficient based on Equation 1 and with respect to hydrogen; the respective definition can be seen in the following Equation 11, all other parameters are described in the equations stated above. As it can be seen in Equation 10, the same yield coefficient *Y*_*H*_2__ is used for all components. The assumption for the experiments is that the yield coefficients are similar for all components.


(11) $$\begin{array}{l} \zeta_{=}\left(\begin{array}{c} -1\\ -\frac{1}{4}\\ \frac{1}{4} \end{array}\right) \end{array}$$

As described in the previous chapter, the main measured value to match for the experiment is the pressure drop inside the reactor. This was done by implicitly computing the components in the water phase, the resulting concentrations in the gas phase, and by applying Equation 8 rearranged to estimate the total gas phase pressure inside the reactor. Thereby the total pressure is the sum of all partial pressures, which are calculated using the ideal gas law and the moles present in the gas phase. The equation system is implemented in Python, and the set of ordinary differential equations is solved by “odeint”, which uses Isoda from the Fortran library odepack.

### 3.2. Initialization and workflow

The workflow for the matching process consists of three steps:(1) initialization; (2) simulation of the base case with base case growth parameters; (3) matching the mass-transfer coefficient, growth rate, and yield coefficient. The main parameter to match is the pressure drop inside the reactor and, if measured, the cell concentration versus time.

Before starting the numerical simulations, the model is initialized. The initial total amount of moles present in the gas phase is calculated by the initial pressure, the gas compositions (80% hydrogen and 20% carbon dioxide) and the gas volume, by applying the ideal gas law. The initial concentrations in the water phase are computed by applying Equation 6 and 7 to correct the operating temperature with Henry's Law and correction coefficients from the literature (Sander, 2015). The used parameters are listed in Table 2. In addition to the initial components in both phases, the microbial density is defined based on one measurement. Thereby, the initial microbial density is calculated by image processing with the help of microscopic pictures. The initial value is approximately 1E7 cells/ml. This value was used in those experiments in which no additional measurements were performed.

**Table 2:** Overview of the constant parameter for the simulation.

**Parameter**	**Value**	**Comment**
*H*_*H*_2__ (*mol*/*m*^3^·*Pa*)	8.58E-06 (at 63°*C*) and 8.32E-06 (at 37°*C*)	Henry constant for hydrogen
*H*_*C*_*O*__2__ (*mol*/*m*^3^·*Pa*)	5.69E-04 (at 63°*C*) and 4.91E-04 (at 37°*C*)	Henry constant for carbon dioxide
*H*_*C*_*H*__4__ (*mol*/*m*^3^·*Pa*)	1.900E-05 (at 63°*C*) and 1.73E-05 (at 37°*C*)	Henry constant for methane
*K*_*C*_*O*__2_, *w*_	0.011 $\frac{mol}{m^{3}}$	Constant for all experiments
		and taken from literature
*K*_*H*_2_, *w*_	0.02 $\frac{mol}{m^{3}}$	Constant for all experiments
		and taken from literature

Sensitivity analysis shows that four kinetic parameters have the most decisive influence on the pressure match and the growth behavior: the magnitude of the yield coefficient, the mass-transfer coefficient, the maximum growth rate and the decay coefficient. During the matching process, those parameters are matched to the pressure and, if available, to the microbial density versus time. It has to be stated that the solution or match is unique only with microbial density measurements. Otherwise, an infinite number of combinations for the matching parameters exist.

The half-saturation constants are kept constant for both species and all experiments, as their influence is minimal. The Table 2 summarizes the parameters for initialization, taken from different literature references (Schönheit et al., 1980; Thaysen, 2021), for species 1 and species 2 in the experiments.

All the other parameters should be determined in matching process. In order to avoid unrealistic values for the matched parameters, the following values are taken as a base case. The values can be found in the literature, however, they are adjusted during the matching process (Schönheit et al., 1980; Thaysen, 2021).

- $\mu_{growth}^{max}$ = 1.11E-4 $\frac{1}{s}$ for species 1 and 3E-5 $\frac{1}{s}$ for species 2
- decay coefficient = 9E-8 $\frac{1}{s}$
- hydrogen yield coefficient (*Y*_*H*_2__) = 5E12 $\frac{1}{mol}$
- initial number of microbes = 1E7 $\frac{cells}{ml}$

The first parameter of the lag phase, *t*_*L*_, is identified when the first pressure drop is measured during the experiments. The second parameter, *t*_*E*_, is the time when half of the pressure drop is reached.

### 3.3. Results of matching workflow

The first aim and step were to match the growth of both species without any modification (refill or increased pressure). The base case values are modified to fit the pressure drop and microbial density for species 1.

**Figure 5:**
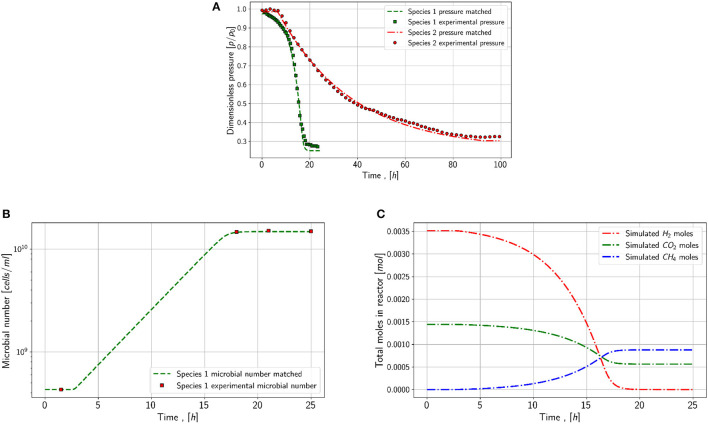
Simulation results for series 1. **(A)** Pressure match for species 1 (green) and species 2 (red) **(B)** Match microbial concentration in the reactor for species 1 **(C)** Simulated moles in the reactor for species 1 during the matched experiment.

As it can be seen in Figure 5A, the simulated pressure for series 1 and series 2 is in alignment with the measured data. The matched maximum growth rate for species 1 is 1.7E-4 $\frac{1}{s}$ and for species 2 1E-5 $\frac{1}{s}$. Both values differ slightly in comparison to the base case values, which differences in species behavior and slight temperature fluctuations in the laboratory could explain. Temperature directly impacts the activity and growth of microbes in the reactor. Based on the changes in the microbial number, shown in Figure 5B, the magnitude of the yield coefficient is matched, and the fine-tuning is made based on the pressure drop. The resulting yield coefficient for species 1 is 4.1E+12 $\frac{1}{mol}$ and for species 2 1E+11 $\frac{1}{mol}$. In contrast, the calculated growth rate has a yield coefficient of high uncertainty, and its correctness could only be validated by comparing the microbial density and gas concentration changes versus time, which is not done. Nevertheless, the magnitude of the yield coefficients is similar to the values from the literature. The parameter set summarized in the following list of species 1 can be stated to be the only possible solution as it matches the microbial density and the pressure at the same time. Also for species 2, other combinations of parameters may be possible, however, the calculated and presented parameters are relatively aligned with the values found in the literature. The matched parameters are summarized in Table 3.

**Table 3:** Matched parameters for the first two experiments.

**Parameter**	**Value**
$\mu_{growth}^{max}$ (1/*s*)	1.7E-4 (SP1) and 1E-05 (SP2)
decay coefficient (1/*s*)	3E-7 for both species
*Y*_*H*_2__ (1/*mol*)	4.1E+12 (SP1) and 1E+11 (SP2)
initial microbial number (*cells*/*ml*)	1.43E7 (SP1) and 1E7 (SP1)

In addition to the pressure and microbial density, the changes in moles in the system are shown for species 1 in Figure 5C. In theory, the behavior of moles in the system is correct, as hydrogen and carbon dioxide decrease and methane increases due to the reaction. It can be concluded that the hydrogen concentration is the limiting factor as it tends faster to zero compared to carbon dioxide.

The mass transfer coefficient for both experiments is similar, 8E-2 $\frac{mol}{s}$ in the experiment with species 1 and 9E-3 in the experiment with species 2. Without the mass-transfer model, the pressure behavior and especially the resulting microbial density are not matchable.

**Figure 6:**
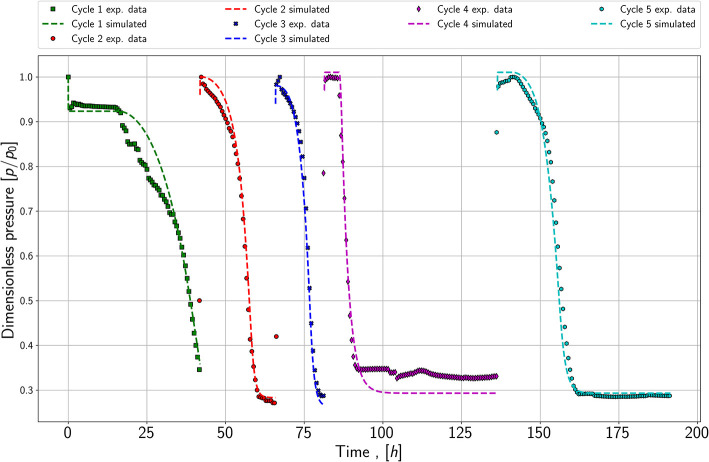
Simulated pressure versus time and experimental data from the refill experiment.

Matching the refill series has a slightly different approach than the previous series. The aim is to keep the growth rate, mass-transfer coefficient, and yield coefficient constant. The initial microbial concentration for each following cycle is the simulated value from the last refill period. As demonstrated in Figure 6 it is possible to match the refill experiment. Thereby the growth rate changes slightly, and the yield coefficients stay nearly constant. The first and initial period is matched with a maximum growth rate of 1.33E-5 $\frac{1}{s}$ and a yield coefficient equals 2.5E+11 $\frac{1}{mol}$. In contrast to the following cycles, the first period has a strong lag phase, which is modeled by setting *t*_*L*_ equals 57,000 *s* and *t*_*E*_ equals 1.35E6 *s*. Further, it can be seen in Figure 6 that the pressure does not reach the stationary phase in the simulation and the measured values. Due to an issue in the laboratory, the refill was performed before reaching the stationary phase, which is also considered in the simulation. To match the first refill or second cycle, the growth rate is increased to 7E-5 $\frac{1}{s}$ and the yield coefficient is set to 2.6E+12 $\frac{1}{mol}$. A significant lag phase was not noticeable; therefore, *t*_*L*_ and *t*_*E*_ are decreased to 60 *s* and 2,400 *s*. The third and fourth refills are matched with similar maximum growth rates: 1E-04 $\frac{1}{s}$ and 1.1E-04 $\frac{1}{s}$. In comparison to the previous cycle, the rates increase again. Further, the yield coefficient stays constant, especially the resulting microbial density. In cycle four, a small lag phase is detectable, which leads to *t*_*L*_ equals 16,800 *s* and *t*_*E*_ equals 33,000 *s*.

The last cycle is matched with the nearly same yield coefficient of 2.5E+11 $\frac{1}{mol}$. However, the cell number at this stage is so high that the growth curve is dominated by the maintenance/stationary phase growth and the substrates are consumed relatively fast compared to the experimental run time. The growth rate decreases slightly to 8E-5 $\frac{1}{s}$, and the lag phase increases again. This could be a result of the microbes remaining longer in the stationary/maintenance phase in cycle 4 and with the new refill, they need to adapt again to the conditions.

The mass transfer coefficient for all cycles stays constant at 5E-2 $\frac{mol}{s}$, which is logical as the contact area between gas and water also remains constant. During the matching process, the key finding was, that either the yield coefficient or the initial microbial density in place had to be matched. Therefore, microbial density initially and at the end of cycle 1 are of great importance. Consequently, the decay coefficient and the initial microbial density are also adjusted for cycle 1. The initial microbial density is decreased compared to experiment one from 1E7 to 5E6 *cells*/*ml* and the decay coefficient is increased to 9E-3 1/*s*. As no microbial density is measured during the experiment, this solution is not exclusive and other combinations may be possible.

### 3.4. Impact of mass transfer coefficient in porous media

**Figure 7:**
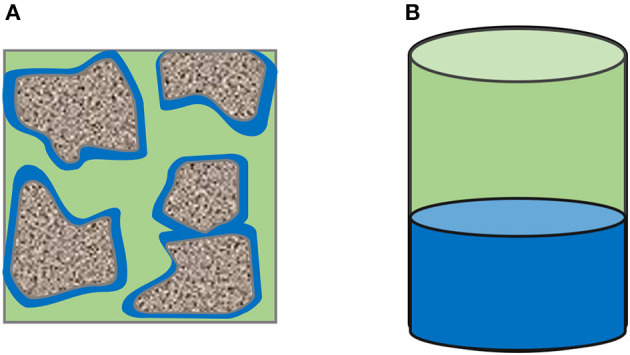
Specific contact area of gas and water in porous media and reactors; green = gas, blue = reactive water. **(A)** Phase distribution inside the porous medium. **(B)** Phase distribution inside the reactor.

As mentioned briefly in the introduction, the results from the performed reactor experiments may differ from reaction rates in porous media. In the syngas-methanation reactor design, porous media and stirred bubble reactor led to a higher methane production due to the increased specific contact area between the gas and liquid phase. A sketch of the liquid distribution for both reactive volumes is illustrated in Figure 7.

In porous media, the specific contact area between gas and reactive liquid depends on the phases' distribution inside the porous media. Due to the liquid film formed around the grains in porous media, the specific contact area significantly increased. In reactors, the gas-water interface depends only on the diameter of the reactor. The mass transfer could be enhanced by stirring or the creation of bubbles at the bottom of the reactor. However, such a reactor design and experimental procedure leads to a complex system which is not the aim of this study.

Nevertheless, with the proposed model it is possible to investigate the impact of an enhanced mass-transfer due to an increased specific contact area, which may be the case in underground hydrogen storage. In order to study the impact of the mass transfer coefficient in porous media, the ratio between contact area and reactor volume needs to be considered. The contact area in the reactor is 1963.4 *mm*^2^ and the reactive volume is 132500 *mm*^3^ which results in a specific surface area of 0.0147 $\frac{mm^{2}}{mm^{3}}$.

Assuming now a total porous sandstone volume as a reactive volume, a residual water saturation of 0.2 and a porosity of 0.35, the specific contact area lies between 45-47 $\frac{m^{2}}{m^{3}}$ based on literature (Cary, 1994; Faisal Anwar et al., 2000; Maalej et al., 2003; Ghiassi et al., 2012). As the mass transfer in the two-film theory depends directly on the specific contact area, the impact of an increased coefficient by a factor of 3200 on microbial conversion can be theoretically simulated. Therefore, the data set from the first series and species 1 is chosen, and the water volume and mass transfer coefficient are both modified to study the conversion in a porous reactor.

**Figure 8:**
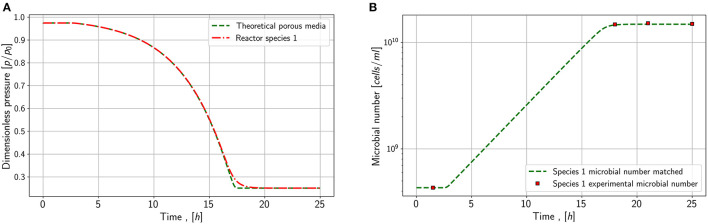
Simulation results for mass transfer study. **(A)** Pressure behavior in a closed porous media and a reactor for species 1 **(B)** Microbial concentration in the reactor and the porous media for species 1.

Figure 8 shows the effect of an increased mass transfer. No strong impact on conversion rates or microbial density can be noticed. Only the shape of the pressure and the microbial density versus time in the later stage of the exponential phase shows slight changes. Water saturation may have an influence as by decreasing the reactive water volume, the substrate amount increases due to an increased gas volume. A combination of increased water saturation and mass transfer coefficient may lead to a higher conversion rate. Besides the specific contact area of gas and water, the mass-transfer depends on the temperature and pressure dependent Henry constant. Consequently, the solubility of the substrates changes with pressure and temperature. Further, the gas flow and the substrate supply could affect the mass-transfer, as the substrate concentration in the gas phase impacts the solubility. Amending the substrate concentration could lead to a higher substrate concentration in the water phase, as the partial pressure of substrate in the gas phase does not decrease.

### 3.5. Model limitations

The proposed model has its limitation. Despite a match, the gas composition changes in the reactor have not been validated in the model. This is required to determine a valid value for the yield coefficient.

Further, the model does not include the influence of temperature on growth rate. It is known that the growth rate of microbes has an optimal temperature, but methanogens can also grow within a specific temperature range. The growth rate has its maximum at the optimal temperature and decreases when the temperature differs from its optimum. To determine the growth under different conditions, a correlation is required, which would make the growth rate dependent on the temperature inside the reactor. The model only considers the effect of temperature on the mass-transfer and the solubility of substrates.

The model only includes the methanation metabolism at this state. Mixed consortia, which would be more realistic in the case of underground hydrogen storage, and the combination of metabolism, e.g., sulfate-reduction and methanation, are not included.

The mass-transfer model depends only on the Henry constants of the substrates, and its model does not consider any diffusion coefficient, which is the main driving flux. This could be implemented in the next stage.

As the experiments are designed to provide optimal conditions and the growth limitation is due to the lack of substrates (*H*_2_ and *CO*_2_), the model can only be used for this case at the moment. Other limitations, e.g. missing trace elements in the water or inhibitors, are not considered. Further, no porous material is added to the reactor, which could also impact the growth.

## 4. Conclusions and recommendations

The study presents a validated numerical model to calculate growth kinetic parameters for the methanation metabolism based on pressure behavior in a batch reactor. Various experiments are performed to test the reactor and collect data for the model process. The proposed mathematical model couples the mass transfer of components between the gas and water phase with the conversion of the substrates and the microbial growth in the liquid phase. A double Monod model with hydrogen and carbon dioxide as limited substrates is implemented to mimic the growth of microbes. The model's main purpose is to calculate growth kinetic parameters like growth rate and yield coefficient. The matched parameters may be used as an input in reservoir simulations to evaluate the impact of microbial conversion during underground hydrogen storage. The model could also be used to predict changing gas composition and the effect of those changes on microbial growth. In combination with the batch experiments, the model could be used to screen storage sites for potential hydrogen conversion due to methanation. Main conclusions can be drawn:

- The proposed model allows to simulate experimental batch reactions, where the substrate is introduced through the gas phase like in an underground hydrogen storage. Further, it could be used to evaluate the growth kinetic parameters and the conversion/consumption rates of hydrogen, which may be important for storage operations.
- Matching the experimental data is achieved by modifying the maximum growth rate, yield coefficient, and mass transfer coefficient. The lag phase model seems suitable to mimic the lag phase observed in the experiments.
- The implementation of a mass transfer model is essential for the matching process. The model still needs improvement to evaluate the effect of increasing temperatures on the mass-transfer, especially on the growth rate itself. With the used model, the impact of a mass-transfer due to a higher specific contact area, which can be found in porous media, is insignificant.

Despite achieving suitable matches, the model has its limitations. At this stage, the model only considers methanation as a conversion process, whereas multiple microbial reactions could take place simultaneously during underground hydrogen storage. Further, the collected data does not contain a representative number of measured microbial density values. Increasing or improving the measured points would lead to a more validated data set of growth parameters. Additionally, the yield coefficients need validation by measuring the gas composition in the gas phase. Adding gas compositional measures to the matching process would improve the understanding of changing yield coefficients during refill phases, comparable to a storage operation.

## Data availability statement

The original contributions presented in the study are included in the article/supplementary material, further inquiries can be directed to the corresponding author.

## Author contributions

GS performed the study and wrote the first draft of the manuscript. All authors contributed to manuscript revision, read, and approved the submitted version.
